# Potential Utility of the SYNTAX Score 2 in Patients Undergoing Left Main Angioplasty

**DOI:** 10.5935/abc.20160038

**Published:** 2016-04

**Authors:** Sérgio Madeira, Luís Raposo, João Brito, Ricardo Rodrigues, Pedro Gonçalves, Rui Teles, Henrique Gabriel, Francisco Machado, Manuel Almeida, Miguel Mendes

**Affiliations:** 1 UNICARV - Serviço de Cardiologia - Hospital de Santa Cruz - Centro Hospitalar de Lisboa Ocidental, Carnaxide - Portugal; 2 Hospital da Luz - Luz Saúde, Lisboa - Portugal

**Keywords:** Angioplasty Balloon Coronary / adverse effects, Coronary Artery Bypass / adverse effects, Myocardial Revascularization, Coronary Artery Disease / surgery, Risk Assessment, Risk Factors

## Abstract

**Background:**

The revascularization strategy of the left main disease is determinant for clinical outcomes.

**Objective:**

We sought to 1) validate and compare the performance of the SYNTAX Score 1 and 2 for predicting major cardiovascular events at 4 years in patients who underwent unprotected left main angioplasty and 2) evaluate the long-term outcome according to the SYNTAX score 2-recommended revascularization strategy.

**Methods:**

We retrospectively studied 132 patients from a single-centre registry who underwent unprotected left main angioplasty between March 1999 and December 2010. Discrimination and calibration of both models were assessed by ROC curve analysis, calibration curves and the Hosmer-Lemeshow test.

**Results:**

Total event rate was 26.5% at 4 years.The AUC for the SYNTAX Score 1 and SYNTAX Score 2 for percutaneous coronary intervention, was 0.61 (95% CI: 0.49-0.73) and 0.67 (95% CI: 0.57-0.78), respectively. Despite a good overall adjustment for both models, the SYNTAX Score 2 tended to underpredict risk. In the 47 patients (36%) who should have undergone surgery according to the SYNTAX Score 2, event rate was numerically higher (30% vs. 25%; p=0.54), and for those with a higher difference between the two SYNTAX Score 2 scores (Percutaneous coronary intervention vs. Coronary artery by-pass graft risk estimation greater than 5.7%), event rate was almost double (40% vs. 22%; p=0.2).

**Conclusion:**

The SYNTAX Score 2 may allow a better and individualized risk stratification of patients who need revascularization of an unprotected left main coronary artery. Prospective studies are needed for further validation.

## Introduction

Unprotected left main coronary artery disease (ULMD) is associated with poor prognosis when medically treated.^1^ Large-scale trials and meta-analysis support that survival is at least similar for both coronary artery by-pass graft (CABG) and percutaneous coronary intervention (PCI) up to 5 years.^2-4^ This consistent non-inferiority has been reflected in the current European revascularization guidelines with PCI of the ULMD being upgraded to a class I and IIa for patients with a low and intermediate SYNTAX (Synergy Between PCI with Taxus and Cardiac Surgery) score, respectively.^5,6^ Nonetheless, selecting the optimal revascularization strategy remains challenging. Despite the inherent strengths and limitations, risk stratification tools are useful as adjuncts for decision-making particularly in the Heart Team setting.^7-10^

The SYNTAX Score 1 (SS1) was created as part of the SYNTAX trial^9,11^ in order to objectively characterize the severity of coronary artery disease (CAD), stratifying patients into low (SS1 <22), intermediate (SS1 23-32) and high (SS1 >33) risk tertiles.^12^ Within this population, the 5-year follow-up supports PCI as an acceptable alternative in patients with ULMD and a low or intermediate risk SS1.^13^ In addition, the prognostic value and usefulness of the SS1 has been extensively studied and substantiated ULMD PCI patients.^14-18^

However some limitations have been pointed out, namely the absence of clinical variables, the lack of a personalised approach to decision-making and the lack of predictive ability in the CABG subset of patients.^8,19-21^

The SYNTAX Score 2 (SS2) emerged to overcome those limitations, by incorporating prognostically important clinical variables and by making an individualised estimate of mortality risk associated with each revascularization strategy.^8^ By applying the SS2 in the all-comers population of the SYNTAX trial it was shown that subsets of patients existed in all tertiles of SS1 in which both CABG and PCI would confer mortality benefit.^8^

We sought to validate and compare the performances of the SS1 and the SS2 as predictors of major cardiovascular events (MACE) at 4 years in patients who underwent ULMD PCI. Furthermore, we aimed to evaluate the long-term outcome according to the SS2 recommended revascularization in a ULMD PCI population.

## Methods

### Patient population and data collection

This was a single-centre, retrospective, observational study that included 132 patients who underwent ULMD PCI between March 1999 and December 2010 with at least one stent implanted in the left main coronary artery. The interventional strategy was left to the discretion of the treating operator. Acceptance of the patient for ULMD stenting required consensus of the Heart Team in the elective cases. All data concerning demographic, clinical, angiographic and procedural characteristics were prospectively entered in our institutional cath lab-based and dedicated database. Post-discharge clinical follow-up was performed during scheduled outpatient visits or telephone interviews. All angiograms were retrospectively analyzed, by two operators blinded for clinical outcomes, for assessment of the angiographic variables necessary for the calculation of the SS1. The SS1 was calculated using the online calculator. The SS2 was estimated manually in each patient for both revascularization strategies (SS2 for PCI and SS2 for CABG) by matching the sum of points of both clinical (age, sex, chronic obstructive pulmonary disease, creatinine clearance, left ventricular ejection fraction and peripheral artery disease) and angiographic variables (SS1 and left main disease) with the corresponding prediction, using the published nomogram.^8^

### Definitions

The left main stem was defined as unprotected if there was no patent bypass graft to the left anterior descending artery or the circumflex artery. Acute myocardial infarction during follow-up was defined according to the 2012 third universal definition of myocardial infarction,^22^ applied retrospectively. Target vessel revascularization and target lesion revascularization were defined as any revascularization procedure of the target vessel or target lesion (from 5 mm distal to the stent up to 5 mm proximal to the stent), respectively. Cardiovascular death was defined as death due to a demonstrable cardiovascular cause or any unexplained death. Stroke was defined as new neurological defect adjudicated by a neurologist based on clinical and imaging features. The primary endpoint (MACE) was defined as the composite outcome of death, nonfatal myocardial infarction, target-vessel revascularization and stroke.

### Statistical analysis

Continuous variables were expressed as means and standard deviation when normally distributed, and as medians and interquartile range when not normally distributed. Normality was tested with the Kolmogorov-Smirnov test and/or Q-Q Plot visual assessment. Discrete variables were expressed as frequencies and percentages. Event-free survival was computed using Kaplan-Meyer estimates.

The performance of the SYNTAX models was analyzed focusing on discriminative power and calibration. Discrimination indicates the extent to which the model distinguishes between patients who will or will not have MACE. It was evaluated by constructing receiver operating characteristic (ROC) curves for each model. The comparison between curves was assessed with the method described by DeLong et al.^23^ Calibration refers to the agreement between observed outcomes and predictions, and was evaluated by using calibration curves and the Hosmer-Lemeshow goodness-of-fit test. Calibration curves were constructed by plotting predictions in the X-axis and the observed outcome in the Y-axis (by decile of the score-derived predictions). Subsequently a linear regression was applied to the plot and a trend line was inferred. The resulting plots allow for a visual comparison between the predicted and the observed probability of the outcome and are characterized by an intercept, which indicates the extent to which predictions are systematically low or high, and a calibration slope, which should be zero in the ideal scenario. The perfectly calibrated predictions stay on the 45-degree line, while a curve below or above the diagonal, respectively, reflects over- and under-prediction, respectively. Furthermore, calibration was tested with the Hosmer-Lemeshow goodness-of-fit test.

The comparison of baseline characteristics and MACE occurrence between patients in whom SS2 favored CABG versus those in whom it favored PCI was performed using the chi-square test or Fisher's exact test, when appropriate, for categorical variables, and the Student *t* test or the Satterthwaite test for continuous variables.

Additionally, the best discriminative value of the difference between SS2 PCI and SS2 CABG for MACE prediction at four years in patients in whom SS2 favoured CABG was determined by c-statistics.

All tests were two-sided and differences were considered statistically significant at a p-value of 0.05. Statistical analysis was performed with SPSS 20.0 software (SPSS Inc., Chicago, IL, USA) and MedCalc version 9.3.8.0 (MedCalc Software, Acacialaan Ostend, Belgium).

## Results

### Baseline clinical, angiographic and procedural variables

The overall baseline clinical, angiographic, and procedural characteristics in the whole population are shown in Table 1.

**Table 1:** Population baseline characteristics

	**Total (n=132)**	**SS_2 PCI>SS2_CABG (n=47)**	**SS2_PCI<SS2_CABG (n=85)**	**p value**
**Baseline characteristics **				
**SYNTAX Score 2 clinical features**				
Age (mean±SD)	66±12	63±14	67±10	0.06
Male sex	105 (79.5%)	25 (53%)	80 (94%)	<0.001
Creatinin clearance (ml/min) (mean±SD)	74±33	69±33	77±32	0.2
Pulmonary chronic obstructive disease	6 (5%)	0	6 (7%)	0.08
Peripheral artery disease	20 (15%)	6 (13%)	14 (16.5%)	0.6
Ejection fraction >50%	93 (70%)	25 (53%)	68 (85%)	<0.001
BMI	26 [24-28.6]	26 [23-29]	26 [24-28]	0.87
Diabetes	35 (27%)	12 (25%)	23 (27%)	1
Dyslipidaemia or statin treatment	92 (70%)	36 (77%)	56 (66%)	0.2
Hypertension on drug therapy	95 (72%)	34 (72%)	61 (71%)	1
Family history of cardiovascular disease	15 (11%)	5 (11%)	10 (12%)	0.54
Smoking (current)	23 (17%)	13 (28%)	10 (12%)	0.03
Previous PCI	43 (33%)	9 (19%)	34 (40%)	0.02
**Clinical setting**				
Stable CAD	70 (53%)	18 (38%)	52 (61%)	0.02
Acute coronary syndrome	61 (46%)	29 (62%)	32 (38%)	0.01
Unstable angina	16 (12%)	8 (17%)	8 (9%)	0.3
Non-ST elevation myocardial infarction	28 (21%)	13 (28%)	15 (18%)	0.2
ST-elevation myocardial infarction	17 (13%)	8 (17%)	9 (11%)	0.3
Cardiogenic shock	9 (7%)	6 (7%)	3 (4%)	0.07
Multi-vessel CAD	62 (47%)	26 (55%)	36 (42%)	0.2
Three-vessel disease	19 (14%)	13 (28%)	6 (7%)	0.003
SYNTAX Score	22 [13.3-32]	29 [18-38.5]	18 [13-26]	<0.001
**Procedure-related characteristics**				
Glycoprotein IIb/IIIa inhibitors	52 (44%)	21 (48%)	31 (42%)	0.6
Drug-eluting stent implantation	95 (72%)	35 (74%)	60 (70%)	0.3
Other vessel PCI	71 (64%)	26 (59%)	45 (61%)	1
Complete revascularization	90 (76%)	26 (66%)	61 (82%)	0.04

SS2: SYNTAX Score 2; PCI: percutaneous coronary intervention; CABG: coronary artery bypass grafting; BMI: body mass index; CAD: coronary artery disease.

The median [interquartile range] SS1, SS2 for PCI and SS2 for CABG were 22 [13.3−31.8], 7.2 [3.5−17.7] and 8.5 [4.6−18.8], respectively. Forty-seven patients (36%) had a SS2 for PCI greater than SS2 for CABG and therefore, theoretically, should preferably have undergone CABG instead of PCI, according to the SS2 recommendation (Table 2).

**Table 2:** SYNTAX Score results

**Score**	**Median (IQR)**
SYNTAX 1	22 [13.3 - 31.8]
SYNTAX 2 PCI	7.2 [3.5 - 17.7]
SYNTAX 2 CABG	8.5 [4.6 - 18.8]
SYNTAX 2 PCI - SYNTAX 2 CABG	-1.1 [-4.3 - 1.4]
SYNTAX 2 PCI > SYNTAX 2 CABG [n (%)]	47 (36%)

PCI: percutaneous coronary intervention; CABG: coronary artery bypass grafting; IQR: interquartile range.

Patients in whom SS2 for PCI was higher than SS2 for CABG (thus favoring CABG) were more likely to be females, smokers, have depressed left ventricular ejection fraction, history of previous PCI, three-vessel disease and presented more often with an acute coronary syndrome (Table 1).

### Four-year outcomes

During the post-procedure 4-year interval, 35 MACE occurred: 13 deaths, 14 repeated revascularization procedures (7 percutaneous interventions and 7 CABG), 4 nonfatal myocardial infarction, and 4 strokes.

**Figure 1:**
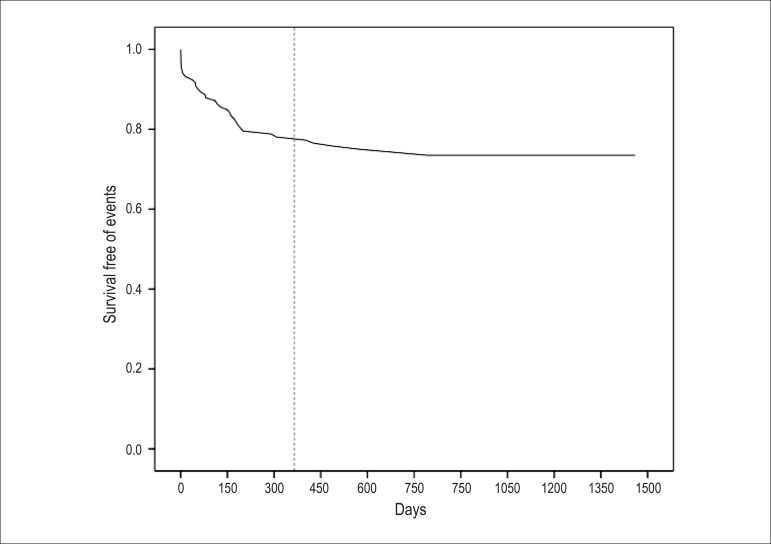
Major cardiovascular event (MACE)-free survival.

The median [interquartile range] time to first event was 117 [25-200] days, with most events (n=28; 80%) occurring during the first year after the index procedure. The cumulative annualized MACE rate was 21%, 26%, 27% and 28% for the first, second, third and fourth years after the intervention, respectively (Figure 1).

### Performance of the SYNTAX 2 models

Because this is a cohort of patients that underwent PCI, we only compared the SS1 with the SS2 for PCI.

### Discriminative Power

**Figure 2:**
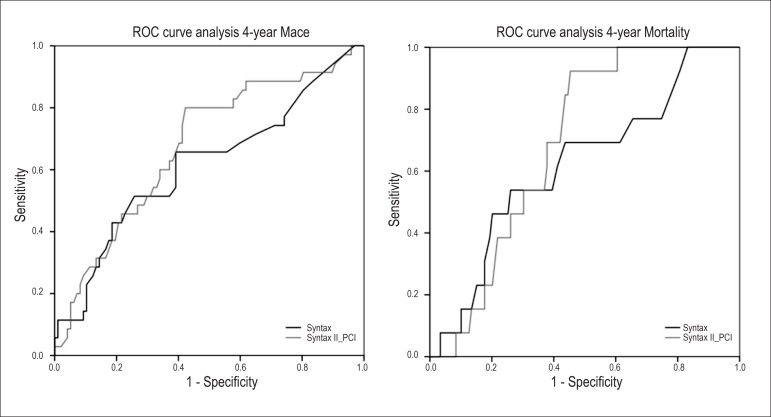
1) SS1 and SS2 ROC curves for major cardiovascular events. (MACE) prediction at 4 years. 2) SS1 and SS2 ROC curves for mortality prediction at 4 years.

With respect to 4-year MACE, the area under the ROC curve (AUC) for the SS1 was 0.61 (95% CI, 0.49-0.73) and 0.67 (95% CI, 0.57-0.78) for the SS2 for PCI (Figure 2). Despite being numerically superior for the SS2, the difference was not statistically significant (DeLong test p=0.08), but there was a relevant trend towards better performance. Concerning 4-year mortality, the AUC for the SS1 was 0.62 (95% CI, 0.46-0.78) and 0.69 (95% CI, 0.59-0.79) for the SS2 for PCI (DeLong test p=0.1).

### Calibration

**Figure 3:**
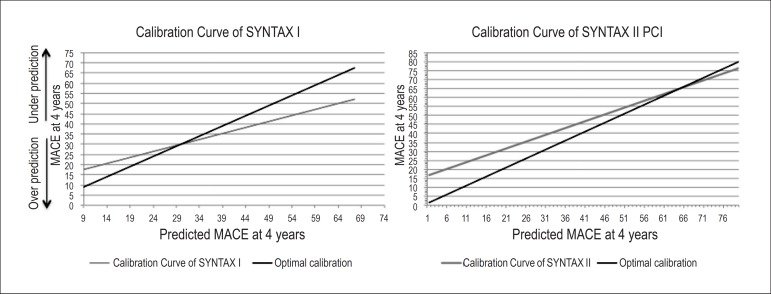
SS1 and SS2 for PCI calibration curves. MACE: major cardiovascular events

The pattern of calibration was different between the two scores (Figure 3): the SS1 tended to underpredict risk in patients at lower risk and to overpredict it in those at high risk. On the other hand, the SS2 for PCI seemed to underpredict risk across almost all risk spectrum, however it gradually approaches the optimal calibration curve as risk increases.

The calibration curve slope and intercept for SS1 and SS2 for PCI are summarized on Table 3. Both scores had nonsignificant p-values (p=0.31 for SS1, and p=0.27 for SS2) for the Hosmer-Lemeshow test indicating that they would provide accurate probabilities.

**Table 3:** Calibration parameters

	**SYNTAX 1**	**SYNTAX 2 PCI**
**Calibration curve**		
Slope	0.59	0.75
Intercept	12.3	15.7
**Hosmer-Lemeshow test**		
p-value	0.31	0,27
Chi-square	9.4	9.9
Nagelkerke R^2^	0.059	0.079

PCI: percutaneous coronary intervention.

### Outcome of patients in whom SS2 would have recommended a different revascularization strategy

Total MACE rate was numerically but nonsignificantly higher in patients in whom the SS2 would have favoured CABG (30% vs 25%; p=0.54) (Table 4).

**Table 4:** Outcomes according to SYNTAX Score 2 recommended revascularization strategy

	**Total (n=132)**	**SS2_PCI>SS2_CABG (n=47)**	**SS2_PCI<SS2_CABG (n=85)**	**p value**
Total MACE	35 (28%)	14 (30%)	21 (25%)	0.5
Death	13 (10%)	6 (13%)	7 (8%)	0.5
**Repeat revascularization**				
CABG	7 (5%)	2 (4%)	5 (6%)	1
PCI	7 (5%)	3 (6%)	4 (5%)	0.7
Myocardial infarction	4 (3%)	2 (4%)	2 (2%)	0.6
Stroke	4 (3%)	1 (2%)	3 (4%)	1

SS2: SYNTAX Score 2; PCI: percutaneous coronary intervention; CABG: coronary artery bypass grafting; MACE: major cardiovascular events.

**Figure 4:**
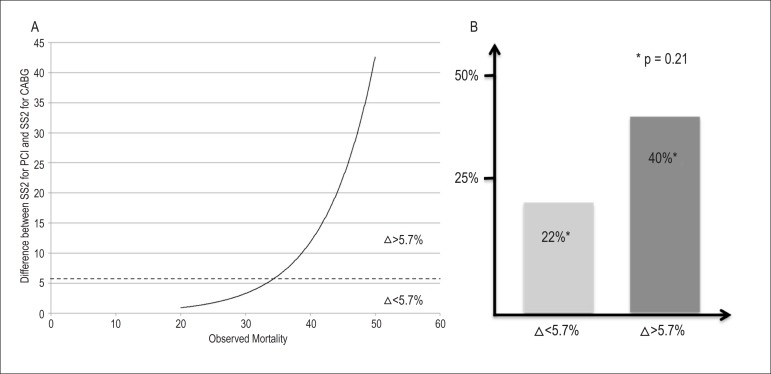
A) Relationship between the absolute difference between the SS2 for PCI and SS2 for CABG with the observed mortality by decile of the difference, in patients in whom SS2 favoured CABG (n=47); B) 4-year MACE in patients in whom SS2 favoured CABG (n=47), stratified according to the ROC-defined best cut-off of the difference between SS2-PCI and SS2-CABG. * p value for the comparison between the values of each column.

To further explore what could be the difference in the scores (SS2 PCI vs. SS2 CABG) that may be clinically relevant, we used the best discriminative value for MACE at 4 years of the difference between SS2 for PCI and SS2 for CABG in the 47 patient subgroup in whom SS2 would have favoured CABG (Figure 4). When the difference was greater than 5.7% (the cut-off value found by ROC curve analysis), MACE rate was almost double (22% vs. 40%); however this difference did not reach statistical significance (p=0.2) (Figure 4).

## Discussion

The main findings of our study were: 1) both scoring systems had a modest performance; 2) overall, the SS2 improved only slightly the performance of the purely anatomic SS1; 3) MACE was nonsignificantly higher in those patients that would have had a different revascularization strategy according to the SS2; and 4) a difference between SS2 PCI and CABG estimates greater than 5.7% may be clinically relevant.

In general, these findings are in line with prior studies assessing the association between the SS1 and clinical outcomes, at different time points,^14-17,21,24-26^ indicating that anatomical complexity alone may be rather insufficient to warrant reliable risk stratification. Although in most of the analysis the overall rate of ischemic events has been systematically higher in patients in the highest risk tertiles,^15,17,24,26^ the discriminative power for mortality and MACE, in both PCI and especially in CABG-treated patients, has been inconsistent. In a population of 949 UMLD cases (400 PCI and 549 CABG), the AUCs of SS1 for 2-year mortality were 0.73 and 0.56 for PCI- and CABG-treated patients, respectively.^19^ In another ULMD cohort (n=1580), the SS1 showed only modest 3-year MACE prediction in patients treated with drug-eluting stents (AUC 0.60), was even worse for patients treated with bare metal stents and CABG (0.48 and 0.51, respectively).^21^ In our study, the AUC of the SS1 for 4-year MACE was 0.61, which is comparable to that shown in other cohorts of ULMD PCI with shorter follow-up (AUCs for SS1 between 0.53 and 0.64).^14,15,21,27^ As in our dataset, others have also shown a poorer discrimination of SS1 for overall composite MACE than for cardiac mortality alone in patients undergoing PCI.^8,14,15,19^

Scarce data exists on the additional value of the SS2. It has been externally validated for long-term mortality in the *Drug Eluting stent of left main coronary artery disease* (DELTA) registry,^8^ and in a large single-centre registry by Xu et al.^28^ that included 1,528 patients with ULMD submitted to PCI. In these cohorts, the SS2 showed an AUC for 4-year mortality of 0.72 and 0.69, respectively, similar to that shown in the original SYNTAX trial population (AUC of 0.73), clearly outperforming the SS1 (AUCs of 0.57, 0.61 and 0.59, in the SYNTAX, DELTA and Xu populations, respectively).^8,28^ Our results concerning mortality also compared favorably to the ones obtained in these larger cohorts: the AUC of the SS1 for 4-year mortality was 0.62 (which is similar to the DELTA registry) and the c-statistic for the SS2-PCI was 0.69 (equal to the reported by the Xu registry^28^ and only slightly lower than the observations in the DELTA registry validation sets). These small differences may be due to a smaller sample size, differences in the rate of the primary endpoint and to the overfitting of the predictive score to its derivation cohort. Recently, the SS2 was prospectively applied to patients included in the *Evaluation of the Xience Everolimus Eluting Stent vs. Coronary Artery Bypas Surgery for Effectiveness of Left Main Revascularization* (EXCEL) trial. It indicated equipoise for long-term mortality between CABG and PCI in subjects with ULMD and intermediate anatomical complexity, and strengthened the notion that both clinical and anatomical features influence mortality predictions.^29^

The Hosmer-Lemeshow test p-value indicated an overall acceptable calibration for both scoring systems; moreover, the SS1 demonstrated a comparable p-value to other registries.^15,27^ The SS1 behaved differently for low- and high-risk patients, underpredicting it in the former and overpredicting in the latter (Figure 3). This kind of performance can theoretically lead to an unrealistic optimism in patients with less risk and at a preposterous concern in those at highest risk. On the other hand, the SS2 tends to underestimate risk progressively less along the spectrum, with the worst performance for low-risk patients and better for high-risk patients. For practical and clinical purposes, the SS2 seems to have a more predictable behavior and therefore should be better suitable for assisting decision-making concerning the optimal revascularization strategy. Overall, as previously outlined, the SS2 performed better (although nonsignificantly) than the SS1 for predicting MACE at 4 years (p=0.08 for the comparison between ROC curves).

It was expected that patients, who should have had CABG instead of PCI according to the SS2 estimates, might have had a higher MACE rate when undergoing PCI. However, despite actually being numerically higher (30% vs. 25%), the difference was not statistically significant. In the Xu et al^28^ registry, which included nearly 10 times as many patients as we did, there was no significant difference in MACE rate between patients that would have had other revascularization strategy according to SS2 (21.6% vs. 24.8%; p=NS).^28^ Still, in all cases it is not known whether patients in either cohort would have had any less MACE if they had undergone CABG instead in the first place. On the other hand, in a pooled analysis of a heterogeneous low-risk profile for a PCI cohort of 5,433 patients enrolled in contemporary coronary stent trials, patients who should have had CABG (less than 1% of all population) according to the SS2 had higher 3-year mortality.^30^ However, in that population, the difference in CAD complexity (assessed by SS1) between the recommended treatment groups was higher than in our cohort. This fact may in part explain the difference found in outcome.

Conceptually, the SS2 would direct the decision between either CABG or PCI on the basis of the estimated risk for each revascularization strategy. The choice would than theoretically "fall" for the strategy associated with the lowest risk. Although this seems to be an intuitive and rational policy, there is no established clinically relevant threshold for the difference between SS2-PCI and SS2-CABG that should mandate a change in strategy. Small and intermediate differences will remain controversial and only large differences will be categorical when deciding the optimal revascularization strategy.

In our cohort of patients undergoing PCI who would have been reclassified for CABG by the SS2, the threshold of the difference between SS2-PCI and SS2-CABG for prediction of MACE was 5.7%. The MACE rate was almost double in those patients with a difference greater than 5.7% (40% vs. 22%). Despite not being statistically significant (analysis of only 47 patients), this finding may be clinically relevant, is surely hypothesis generating, should be explored in larger cohorts including patients submitted to both CABG and PCI, and, if confirmed, validated prospectively in a clinical trial.

### Limitations

Some important limitations should be pointed out in our study. First, the inherent limitations of a single-centre retrospective study. Second, the limited number of patients may have limited the power of the statistical analysis and the ability to find statistical significance for many of the comparisons. Third, the long time span of the registry (~10 years) renders the group highly heterogeneous, especially considering that a significant number of patients treated with bare metal stents was included. This goes against contemporary practice in ULMD PCI and is in marked contrast with the original SYNTAX trial cohort, in which TAXUS stents were used, and from which the original scores have been derived. Fourth, our analysis did not take into account the location of the lesions in the left main coronary artery and the different stenting techniques for distal and bifurcation lesions. Not only have there been variations in the stenting strategies throughout the study period, but these also play a role in defining the complexity and success of the procedure and would help to interpret our results. However, in our cohort of ULMD patients, lesion location within the left main coronary artery was not an independent predictor of 5-year MACE,^31^ and Capodano et al.^18^ have not found a prognostic impact of the stenting technique, regardless of the baseline SS1. Fifth, it is not possible to ascertain the extent to which confounders inherent to specific selection criteria for left main stenting have influenced MACE rates and thus the predictive ability of the scores, especially if we bear in mind that a large part of this population was included at a period when CABG would be regarded as a more common choice. Finally, true validation of SS2 would require random assignment to either CABG or PCI in a prospective study.

## Conclusions

The SYNTAX Score 2, by combining and weighting clinical and anatomical features, may allow a better and individualized risk stratification of patients who need revascularization of an unprotected left main coronary artery. A difference greater than 5.7% between SYNTAX Score 2 estimates for PCI versus CABG may be clinically relevant in selecting the optimal revascularization strategy. Prospective studies are needed for further validation.
